# Impact of Data Capture Methods on 3D Reconstruction with Gaussian Splatting

**DOI:** 10.3390/jimaging11020065

**Published:** 2025-02-18

**Authors:** Dimitar Rangelov, Sierd Waanders, Kars Waanders, Maurice van Keulen, Radoslav Miltchev

**Affiliations:** 1 Technologies for Criminal Investigations, Saxion University of Applied Sciences, 7513 AB Enschede, The Netherlands; s.c.waanders@saxion.nl (S.W.); k.t.waanders.01@saxion.nl (K.W.); 2 Faculty of Electrical Engineering, Mathematics and Computer Science, University of Twente, 7522 NB Enschede, The Netherlands; m.vankeulen@utwente.nl; 3 Police Academy of The Netherlands, 7334 AC Apeldoorn, The Netherlands; 4 Faculty of Industrial Technology, Technical University of Sofia, 1756 Sofia, Bulgaria; rmiltchev@tu-sofia.bg

**Keywords:** 3D reconstruction, neural radiance fields, gaussian splatting, 3D scanner technology, crime scene reconstruction, forensic photogrammetry, forensics

## Abstract

This study examines how different filming techniques can enhance the quality of 3D reconstructions with a particular focus on their use in indoor crime scene investigations. Using Neural Radiance Fields (NeRF) and Gaussian Splatting, we explored how factors like camera orientation, filming speed, data layering, and scanning path affect the detail and clarity of 3D reconstructions. Through experiments in a mock crime scene apartment, we identified optimal filming methods that reduce noise and artifacts, delivering clearer and more accurate reconstructions. Filming in landscape mode, at a slower speed, with at least three layers and focused on key objects produced the most effective results. These insights provide valuable guidelines for professionals in forensics, architecture, and cultural heritage preservation, helping them capture realistic high-quality 3D representations. This study also highlights the potential for future research to expand on these findings by exploring other algorithms, camera parameters, and real-time adjustment techniques.

## 1. Introduction

The introduction should briefly place this study in a broad context and highlight why it is important. Three-dimensional (3D) reconstruction creates detailed digital models of real-world objects or scenes using 2D images, providing precise spatial data for fields like engineering, medicine, and archaeology [1]. One area that could greatly benefit from this technology is crime investigation, where reconstructing crime scenes help investigators understand what occurred. The challenge lies in interpreting the aftermath as evidence which often offers multiple possible scenarios. To develop a complete narrative, the investigators must combine physical traces with additional information such as police reports, witness accounts, forensic data, and their own expertise.

The reconstruction process relies on key principles such as achieving adequate image overlap, minimizing motion blur, and ensuring consistent lighting, all of which are critical for producing high-quality models [2]. While extensive research has been conducted on 3D reconstruction algorithms, the literature on image acquisition methods remains limited, particularly for constrained indoor environments like crime scenes. Existing studies emphasize the significance of camera movement and stability for enhancing reconstruction fidelity, especially in settings with limited space or intricate spatial layouts [3,4]. Moreover, approaches tailored for indoor reconstructions, such as photogrammetry-based workflows and structured light systems, have demonstrated varying degrees of success [5,6,7,8].

Traditional photogrammetry remains a widely used method in 3D reconstruction, particularly for forensic and architectural applications. It relies on structure-from-motion (SfM) [9] and multi-view stereo (MVS) [10] techniques to generate 3D models from 2D images. While photogrammetry provides accurate reconstructions with measurable geometric fidelity, it often requires an extensive manual effort for camera calibration, feature matching, and mesh generation. Additionally, photogrammetric methods struggle in scenes with low-texture surfaces, reflective materials, or non-uniform lighting conditions, leading to incomplete reconstructions and increased noise. These limitations pose significant challenges in forensic applications, where scene complexity and environmental variability must be accounted for.

Recent advancements in deep-learning-based reconstruction, such as Neural Radiance Fields (NeRF) [11] and Gaussian Splatting (GS) [12], present alternatives that address some of these challenges. Unlike photogrammetry, NeRF synthesizes new viewpoints by learning a continuous volumetric representation of the scene, allowing for superior handling of complex lighting and occlusions. However, NeRF is computationally demanding and requires long training times, making it less practical for time-sensitive forensic reconstructions. Gaussian Splatting, on the other hand, offers a more efficient approach by representing 3D structures as a set of discrete Gaussian functions, enabling fast and high-fidelity rendering while reducing manual processing efforts. While both methods improve visual quality and automation, their suitability for forensic applications depends on balancing accuracy, processing efficiency, and real-world usability.

In this research, the focus is on optimizing the data acquisition process for 3D reconstruction in indoor crime scenes by evaluating filming parameters such as camera orientation, operator speed, and path selection. These parameters were chosen due to their fundamental impact on scene coverage, motion blur reduction, and data alignment, critical factors for achieving high-fidelity reconstructions in constrained forensic environments. While factors like lighting conditions, exposure settings, and camera calibration influence reconstruction quality, they are either hardware-dependent or have been extensively explored in prior research, whereas filming methodologies remain an underexplored yet crucial aspect of 3D reconstruction optimization. Examining how the method of filming a crime scene impacts these reconstructions’ quality provides valuable insights not only for forensic applications but also for other fields. For example, architectural visualization can benefit from accurate indoor 3D reconstructions for design and renovation projects [13,14,15]. In cultural heritage preservation, detailed reconstructions of historical sites and artefacts can aid in documentation and restoration efforts [16,17]. Similarly, virtual reality (VR) and augmented reality (AR) applications in gaming and simulation training can achieve higher realism and immersion with optimized 3D reconstruction techniques [18].

To achieve improvements in 3D reconstruction, Neural Radiance Fields (NeRF) [11] is utilized. NeRF is an innovative approach that employs deep learning to generate highly detailed and accurate 3D models from 2D images. It achieves this by modeling the complex interplay between spatial geometry and light behavior in the scene, capturing fine details such as texture and reflectance. NeRF’s ability to synthesize realistic views from novel perspectives makes it particularly suitable for applications demanding precision and realism. By utilizing volumetric rendering, NeRF constructs continuous 3D representations, which are further enhanced when combined with complementary techniques such as Gaussian Splatting, to produce models with superior visual fidelity and minimized artifacts. Gaussian Splatting further enhances this process by refining and smoothing the spatial data points, leading to better-quality reconstructions. Additionally, 3D Gaussian Splatting (3D-GS) [12] is a promising technique in computer graphics for 3D rendering of scenes, gaining traction due to its ability to efficiently render high-quality images while maintaining a compact scene representation. Unlike NeRF, which relies on neural networks conditioned on viewpoint and position, 3D-GS employs Gaussian functions that can be rasterized directly into images, facilitating faster rendering speeds and improved visual fidelity. Similar Gaussian-based methods have been successfully applied in broader fields, such as data assimilation for environmental modeling and field reconstruction, where they help interpolate sparse observations and reconstruct dynamic spatial fields [19,20]. These methods demonstrate the versatility of Gaussian-based approaches in various applications, reinforcing their potential in 3D reconstruction.

NeRF is not the first step in this new evolution of 3D reconstructions but is, rather, the building block of a family of algorithms, which includes SNeRF [21], Tetra-NeRF [22], NeRFacto [23], Instant-NGP [24], SPIDR [25], MERF [26], and so on. In fact, each one of them solves particular problems and allows for the increment of overall capabilities in different ways.

Preliminary research highlighted the importance of the camera, lens, and their settings as critical factors. The impact of each parameter on 3D reconstruction was thoroughly examined [27,28]. Additionally, the method used to film an environment was found to have a significant influence on the quality of the 3D reconstruction. Building on these findings, specific camera movement techniques were employed to ensure comprehensive scene coverage and high-quality data acquisition.

This paper concentrates solely on the method of filming indoor environments. The experimental setup consists of a living room, kitchen, and hallway. Four comparison experiments were conducted, varying one parameter at a time, to identify the optimal scannig method for high-quality 3D reconstructions in indoor crime scene scenarios.

We investigate various factors that influence the quality of 3D reconstructions, focusing primarily on camera orientation, operator walking speed, layering techniques, and scanning paths. The orientation of the camera during capture plays a significant role. Landscape mode offers a broader horizontal field of view, ideal for wide scenes, while portrait mode enhancing vertical detail, which is better suited for tall subjects. Capturing the same environment in both modes enables an analysis of differences in data quality, level of detail, and the accuracy of 3D reconstructions.

Another key factor is the walking speed of the camera operator, as rapid movement can cause autofocus issues, resulting in blurry frames that negatively impact the reconstruction quality. By testing various walking speeds and comparing the resulting video lengths, we assess how motion clarity influences the final 3D model. Furthermore, the use of various layering techniques is examined to enhance data capture from multiple heights, along with the significance of scanning paths to achieve comprehensive environmental coverage. Together, these tests aim to optimize the methodology for achieving high-quality 3D reconstructions.

Accurate 3D reconstructions in crime scene investigations may provide crucial insights into the sequence of events and assist investigators in analyzing evidence more efficiently. The traditional way of representation, like sketching or photographing the scene, neglects important details. On other hand, 3D reconstruction provides an overall interactive way to view the scene. This technology aids in visualizing the crime scene, understanding the spatial relationships between various pieces of evidence, and displaying discoveries in court.

In this paper, the main contributions are provided to the field of 3D reconstruction with a special emphasis on its application within crime scene investigation. The contributions of research can be divided into theoretical and practical advancements.

Theoretical Contributions

- Novel framework for filming method optimization: This study introduces a structured framework for systematically analyzing and optimizing key parameters, camera orientation, filming speed, camera layers, and filming path, for 3D reconstructions. While based on existing methods, this framework uniquely addresses the specific challenges of indoor forensic environments, such as limited space, variable lighting, and object clutter, which have not been comprehensively addressed in prior research.
- Extension of theoretical framework: This research extends the theoretical framework related to 3D reconstruction. For example, a theoretical framework for cameras to be optimized could be any application that it has not seen before. There is big potential of using advanced techniques in reconstruction for architecture, archaeology, and digital media fields.

Practical Contributions

- Crime Scene Investigation Guidelines: The guidelines in the research demonstrate how forensic investigators can achieve an accurate 3D reconstruction of a crime scene. The primary focus of this paper was on the methodology for filming a crime scene. The key takeaway is the importance of capturing the scene from multiple angles by circling around specific objects in different zones.
- Application of advanced 3D reconstruction techniques: This study examines the use of Gaussian Splatting within existing 3D reconstruction workflows, focusing on its effectiveness in handling complex scenes. These include environments with intricate spatial layouts, dense object arrangements, and varied textures, which are particularly challenging in forensic applications. While no modifications to the underlying algorithms were made, this research contributes by detailing how these techniques can be systematically applied and optimized to address the unique challenges of reconstructing detailed, accurate models of such environments.
- 3D Modelling Processes Optimization: The results of the research give very practical recommendations for the optimization of 3D reconstruction processes within numerous fields. These recommendations can significantly improve the quality of 3D reconstructions used in architectural visualization, cultural heritage preservation, and VR/AR applications for enhanced realism and immersion.

This paper introduces significant advancements in the methodology of 3D reconstructions for crime scene investigations by combining state-of-the-art techniques, such as Neural Radiance Fields and Gaussian Splatting, with novel optimization strategies tailored for forensic applications. The research offers innovative insights into how filming parameters, such as camera orientation, walking speed, layering, and scanning paths, can be systematically optimized to achieve higher-quality reconstructions. These contributions not only enhance existing methodologies but also provide practical guidelines for real-world forensic scenarios, thereby expanding the scope of 3D reconstruction technology across various professional domains.

This paper is organized as follows: Section 2 describes the methodology used in this study, including the methods employed during image capture and how they meet the demands of crime scene investigations. Section 3 presents the results of our experiments and comparative analysis of different methods. Section 4 provides a detailed discussion of these findings, comparing them with the existing literature and exploring their implications for various applications. Finally, Section 5 concludes this paper, summarizing our key contributions and suggesting directions for future research.

## 2. Materials and Methods

### 2.1. Experimental Setup

**Figure 1:**
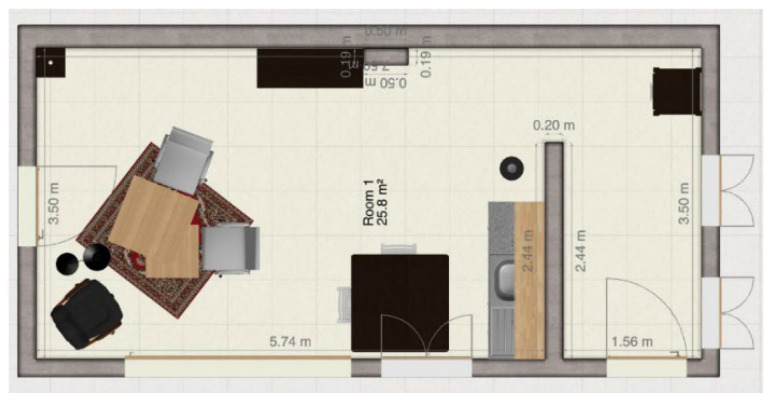
Experimental setup.

The experimental setup consists of a living room, kitchen, and hallway with several key features, such as tables, chairs, tableware, closets, ceiling, and lamps. These elements were chosen to provide a diverse set of objects with different geometric and textural information for the 3D-GS. The floor plan of the room is displayed in Figure 1, which illustrates the arrangement of the environment used in the experiments.

The primary equipment that was used was a SONY a7c, Sony Corporation, Tokyo, Japan [29] camera with a 24.2 MP full-frame Exmor R CMOS BS, Sony Corporation, Tokyo, Japan [29]. This camera was chosen for its full-frame sensor, which results in high image quality. The lens combined with the camera is a Sigma, Sigma Corporation, Kanagawa, Japan 14 mm f/1.4 DG DN [30] Art lens, which is a wide-angle lens. With this lens, capturing more of a room in single capture is possible. To create an even more consistent capturing method, a DJI RS 4,DJI, Shenzhen, China [31] gimbal was added to the camera setup. The gimbal will result in a smoother capture of the environment, with less vibration and thus, fewer unclear frames. Controlled lighting conditions were maintained across all captures.

To improve the quality of 3D reconstructions, particularly in noise reduction, and detail accuracy, Postshot [32] was utilized, an end-to-end software for Radiance Fields to generate the 3D-GS. The goal was to optimize the capturing method of rooms such as mentioned above, while generating accurate 3D models.

In this implementation, Gaussian Splatting was employed to enhance scene geometry representation using a set of 3D Gaussian functions. These functions were rasterized directly into 2D images, which allowed for efficient rendering while preserving high visual fidelity. The Postshot software automates key aspects of this process but provides options for manual parameter adjustments to fine-tune results. For this study, we utilized a Splat ADC profile, which optimizes spatial data distribution by regularizing density values, thereby reducing artifacts and enhancing fine details.

Key parameters included a downsample resolution of 1600 pixels for the input images and a splat density adjustment layer set to 1. These settings were calibrated through iterative testing to balance computational efficiency and reconstruction quality. The training process was configured to stop at 54 kSteps, based on convergence criteria observed during early experimentation.

The integration with NeRF involved aligning Gaussian splats with the neural radiance field’s density and color predictions. Camera poses were computed from the captured images using Postshot’s internal algorithms, ensuring accurate alignment of spatial data with radiance field outputs. By combining NeRF’s volumetric rendering and Gaussian Splatting’s spatial refinement, the method achieved improved clarity and reduced noise in the final 3D models.

**Figure 2:**
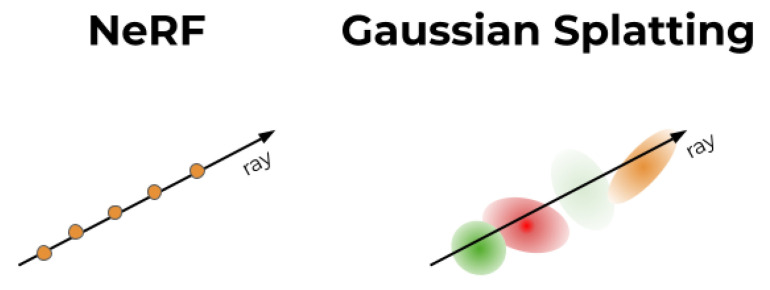
A conceptual difference between NeRF and GS, **Left**: Query a continuous MLP along the ray, **Right**: Blend a discrete set of Gaussians relevant to the given ray [34].

A key distinction between NeRF and Gaussian Splatting lies in how they represent and process 3D scene information as shown in Figure 2. NeRF constructs a scene using a volumetric neural representation, where the space is densely populated with points that encode density and color information. These points do not exist as discrete entities but are instead sampled through a neural function that maps spatial coordinates to radiance values. Rendering a scene requires ray-marching through this continuous representation, accumulating color and transparency at each sampled point along a given viewing direction. While this method produces highly detailed and photorealistic reconstructions, it is computationally expensive and struggles with real-time performance.

In contrast, Gaussian Splatting represents the scene as a discrete set of 3D Gaussian functions (‘splats’) [33], each carrying spatial position, opacity, and shape properties. Unlike NeRF’s volumetric points, which require extensive neural computation to render, splats are directly projected onto the image plane, allowing for rasterization-based rendering that is significantly faster and more efficient. Furthermore, the overlapping and blending nature of Gaussian splats helps to fill in missed spots and smooth out inconsistencies in areas with sparse input data, reducing gaps that might otherwise appear in the reconstruction.

For this research, Gaussian Splatting was chosen over NeRF’s point-based volumetric representation due to its ability to produce faster and cleaner reconstructions. By using splats instead of a densely sampled neural point cloud, our method achieves clearer object boundaries, fewer artifacts, and improved texture fidelity, which is essential for forensic investigations. This choice ensures that reconstructions remain both high-quality and practical for real-world applications where clarity and efficiency are critical.

We conducted four comparison experiments, each varying one parameter of capturing at a time, using the same optimal camera setting and setup during all comparisons. The video footage were processed into 3D reconstructions, which were evaluated based on noise and detail accuracy. These criteria were chosen for their importance in forensic applications where both clarity and realism are essential for analyzing evidence.

This approach allowed us to identify the optimal scan method for high-quality 3D reconstructions in indoor crime scene scenarios, providing valuable insights for future forensic investigations.

### 2.2. Camera Settings

Various camera settings significantly influence the quality of a 3D reconstruction. This study does not focus on camera settings. Therefore, the camera is set to automatic mode. One parameter that is not set in automatic mode is the aperture. A higher aperture results in a more detailed background [35]. The aperture is set to f/5.6 during the capturing, which is the maximum aperture of the Sony a7c.

### 2.3. Data Collection

#### 2.3.1. Capturing Techniques

The camera was handheld with a gimbal throughout the capture process, allowing for flexible, controlled, and stable movements as needed. The selection of capturing techniques was guided by their ability to maximize scene coverage, minimize occlusions, and enhance reconstruction fidelity in forensic applications. In indoor crime scenes, capturing a comprehensive and accurate dataset is crucial, as missing or distorted details can compromise forensic analysis. The chosen techniques, truck, pedestal, boom, and arc were selected due to their effectiveness in maintaining smooth motion, consistent framing, and minimizing abrupt perspective shifts that could degrade reconstruction quality. These techniques offer a structured approach to data acquisition, ensuring that all key objects and spatial relationships within the crime scene are accurately represented [28]. Each technique was strategically used at different points along the paths based on the spatial characteristics of the scene and the specific details we aimed to capture. Other potential methods, such as static multi-angle photography or robotic scanning, were considered but deemed less practical due to their increased setup time and limited adaptability to dynamic environments. By integrating these controlled capturing movements, this study aims to provide an optimized and reproducible approach to filming for high-quality 3D reconstructions.

These techniques were not used for comparison but combined into the different capturing methods to complement each other during the capturing process. Each technique was applied based on the specific spatial requirements of the scene.

#### 2.3.2. Scanning Methods Comparison

Orientation Impact on 3D Reconstruction Quality

This test examines the impact of landscape and portrait modes on 3D reconstruction. Landscape mode (horizontal orientation) captures wide scenes, providing a broader field of view and more horizontal overlap between frames. This will ensure comprehensive scene coverage. Portrait mode (vertical orientation) is ideal for tall objects, offering better vertical detail with more overlap on the vertical axis. By capturing the same environment in both modes, the differences in data quality, level of detail, and accuracy of the 3D reconstructions were assessed.

Effect of Walking Speed on 3D Reconstruction Quality

During filming, the camera operator navigates through the environment to collect data. If the camera operator moves too quickly, some video frames may become blurry because the autofocus cannot keep up. This test aims to examine the impact of blurry frames on 3D reconstruction. The difference in movement speed was assessed by comparing video lengths. Specifically, the duration of the video recorded at a slower movement speed is expected to be twice as long as the video recorded at a faster movement speed.

Layering Technique for Enhanced 3D Reconstruction

**Figure 3:**
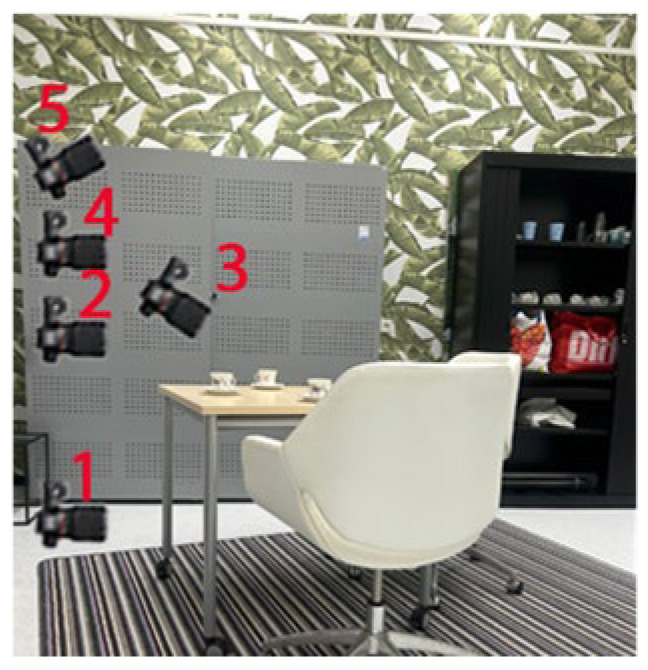
Layering technique, the numbers indicate the position of the layers.

Based on preliminary research, the importance of capturing an environment at various heights has been identified. For methods 1 to 10, we utilized a 3-layer technique, with cameras positioned at 0.3 m, 1 m, and 1.7 m. This approach allows for more comprehensive data capture, leading to a better overall picture of the environment and improved 3D reconstruction. However, capturing the environment with more layers does increase the time required. The test aims to examine the difference in 3D reconstruction quality when capturing the environment with 1, 3, or 5. The same object/zone was filmed using identical settings and setup, but the number of layers was varied. The 5 passes that have been taken are shown in Figure 3. For 1 pass we used camera pose 3, for 3 passes we used 1, 3, and 4, for 5 passes we used all 5 of them.

**Scanning Paths**

The previously mentioned parameters play a crucial role in data collection. Another significant factor is the specific path taken through an environment. Each path differs in some way from each other. The results from testing the different filming paths were a base for the next paths. This continued until there was a suitable path for the use case. All the paths have been filmed with the same camera with the same settings. The different scanning paths are captured with the optimal film technique which are concluded from tests 1, 2, and 3.

Below the scanning paths are displayed and shortly explained:

**Method 1: Following the contours of the whole environment**

**Figure 4:**
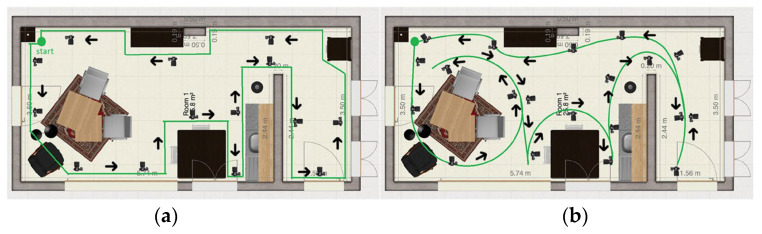
(**a**) Method 1: Following the contours of the whole environment, (**b**) Method 2: Circle around the main objects in the rooms in one path.

The camera operator follows the contours of the environment while pointing the camera inwards. The path is shown in Figure 4a. The camera operator starts in the upper left corner.

**Method 2: Circle around the main objects in the rooms in one path**

The camera operator follows circles partly around the main objects in the room in one connected path. The objects are a dining table, kitchen table, kitchen, closet, and hallway. This method is expected to create more detail of the individual objects because the focus was more on that. The path is shown in Figure 4b. The camera operator starts in the upper left corner.

**Method 3: Circle around the main objects in the room and separate hallway**

**Figure 5:**
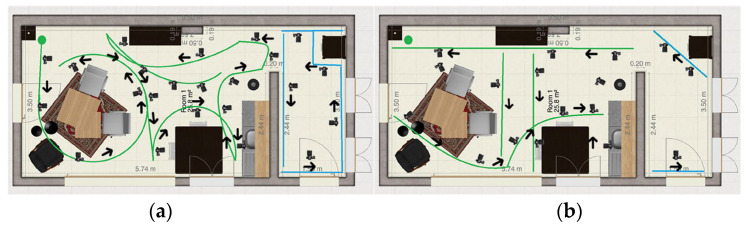
(**a**) Method 3: Circle around the main objects in the room and separate hallway, (**b**) Method 4: Four passes in the living room and separate hallway.

This method is similar to method 2, the main difference is that the hallway is filmed separately from the kitchen/living room. At the end of the path in the living room, there is an extra part added to obtain more data from the room from a distance. The path in the hallway follows the same principle as in method 1. The two videos are combined in the 3D reconstruction algorithm separately to create two reconstructions. The paths are shown in Figure 5a.

**Method 4: Four passes in the living room and separate hallway**

The camera operator makes two separate passes while facing the camera inward on the horizontal axis. On the vertical axis in the living room, the camera was facing outwards. In the hallway, the cameraman should make two horizontal passes on either side while facing the camera inwards. The paths are shown in Figure 5b.

**Method 5: Individual objects**

**Figure 6:**
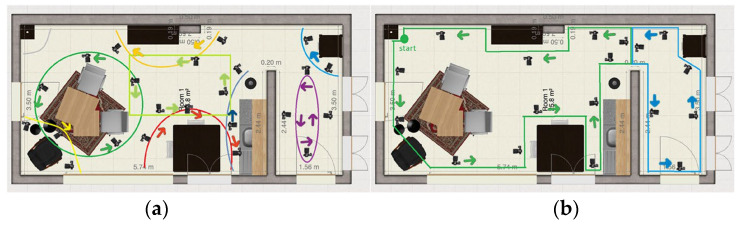
(**a**) Method 5: Individual objects, (**b**) Method 6: Alternative to Method 1.

Every main object/zone will be filmed separately to create more detail. The different paths are shown in the image to the left. The path is shown in Figure 6a. The room is separated in the following objects/zones: leather seat, side table, dining table, kitchen table, closet, kitchen, corner closet, and hallway. With this method, the videos will be put in the 3D reconstruction algorithm separately.

**Method 6: Alternative to Method 1**

This method is the same as method 1 but now the living room and the hallway are separated. The two videos will be put in the 3D reconstruction algorithm separately. The path is shown in Figure 6b.

**Method 7: Three-zone scans**

**Figure 7:**
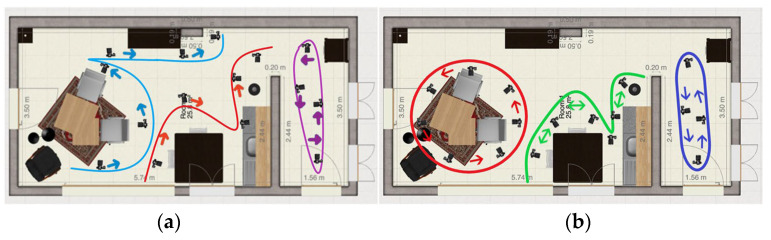
(**a**) Method 7: Three-zone scans, (**b**) Method 8: Three-zone scans v2.

The room is separated into three zones; each zone was captured separately. The path is shown in Figure 7a. Zone one (blue) captures the living area, zone two (red) captures the kitchen area, and the third zone (purple) captures the hallway. These three zones/videos will each be put in the 3D reconstruction algorithm separately.

**Method 8: Three-zone scans v2**

It was found in the previous testing round that the best results were obtained when the room was divided into multiple zones. In Method 8, as shown in Figure 7b, the setup involves two loop closures: one at the coffee table and one in the hallway.

**Method 9: Two zone scans**

**Figure 8:**
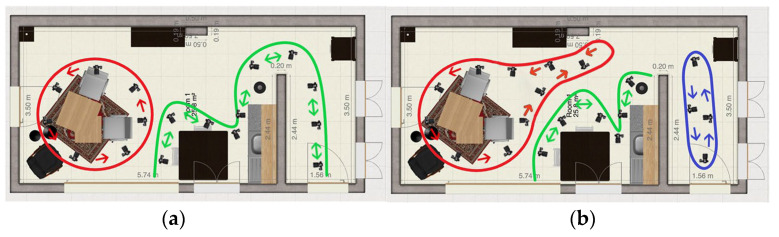
(**a**) Method 9: Two zone scans, (**b**) Method 10: Alternative of Method 8.

Method 9 involves capturing two videos and is presented in Figure 8a. One circles around the coffee table, while the other follows a continuous path around the dining table, kitchen, and hallway. This approach is expected to improve capture of the hallway. However, it may overlook some details, specifically the windows and the closet in the hallway corner. To address this, it is important to focus on capturing these features when rounding the corner.

**Method 10: Alternative of Method 8**

This method is similar to method 8; the only difference is the extra loop around the closet. This path is shown in Figure 8b. This method is expected to have a better reconstruction of the black cabinet.

### 2.4. Comparative Analysis Criteria

In this study, we captured a continuous video sequence using a handheld camera, which was then processed into a 3D reconstruction model. The 3D reconstruction is assessed using certain viewpoints in the 3D reconstruction. These viewpoints were chosen for their ability to represent different textures, lighting conditions, and surfaces common in indoor crime scenes.

There are three focus areas: the living room with a coffee table, the kitchen with a dining table, and the hallway. These areas offer varying textures and lighting challenges, making them ideal for comparison. The 3D reconstructions were evaluated based on three criteria: (1) Noise (artifacts in the model), and (2) Details (fidelity of the reconstruction), (3) Splats. In Table 1, these criteria are rated on a 1–5 scale, allowing us to systematically compare different scanning methods. This framework adds a novel approach for assessing 3D reconstructions in forensic applications.

## 3. Results

### 3.1. Orientation Impact on 3D Reconstruction Quality

**Figure 9:**
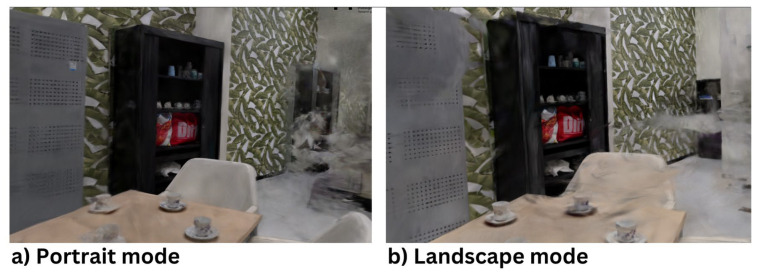
A comparison of 3D reconstructions in (**a**) Portrait mode, and (**b**) Landscape mode. The portrait mode reconstruction shows a duplicated black closet artifact, extending beyond the actual scene. This anomaly is absent in landscape mode, which provides better spatial accuracy by ensuring more horizontal overlap for feature matching.

The purpose of this test is to evaluate the difference in the quality of 3D reconstructions between environments captured in portrait mode and landscape mode. During the scanning process, Method 6 is employed. The difference between portrait and landscape modes is slight but noticeable. As illustrated in Figure 9, the quality is generally comparable. However, in portrait mode, the wall with leaves exhibits a duplicated black closet, and the wall continues beyond this duplicated closet. This anomaly is absent in landscape mode.

Additional scans comparing the two orientations consistently showed that landscape mode yields slightly better results. The primary conclusion is that filming in landscape mode is superior to portrait mode. We hypothesize that portrait mode is slightly inferior because most data overlaps on the vertical axis rather than the horizontal axis. Given that the camera operator moves along the horizontal axis, horizontal overlap is preferable to ensure features are recognized and matched more frequently.

### 3.2. Effect of Walking Speed on 3D Reconstruction Quality

The duration of the video recording determines the velocity of the camera operator. The trajectory followed by the camera operator remains consistent across both videos. However, the speed at which the operator moves varies. This study evaluated two walking speeds: slow speed at 0.085 m/s and normal speed at 0.164 m/s. The speed velocity was measured by Garmin Fenix 6 Pro Solar [36] and later verified by calculation. The duration of the video recorded at normal speed (3:43 min) is approximately twice as long as that of the faster video (1:56 min).

**Figure 10:**
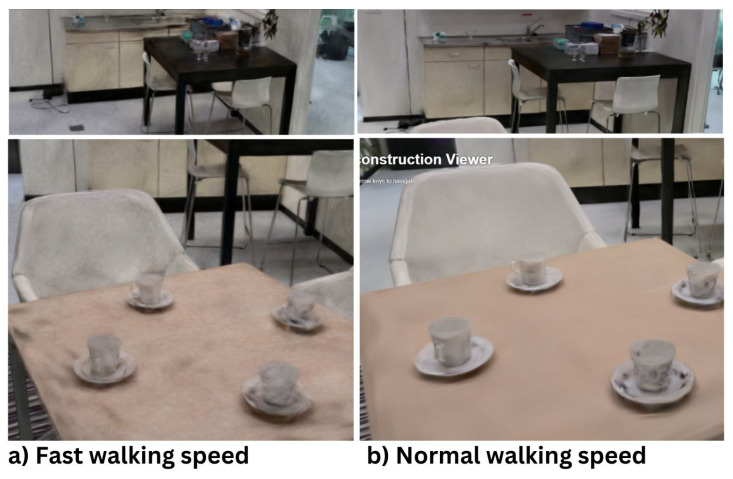
(**a**) Fast walking speed, (**b**) Normal walking speed.

The differences between the fast and slow recordings are clearly evident. At a slower speed, more details, particularly smaller objects, become visible. Additionally, there is significantly less noise in the 3D reconstructions produced from the slower video. This experiment demonstrates that slower filming speeds enhance the detail of the reconstruction. The underlying reason is that a longer video provides more frames for the algorithm to select from. Furthermore, slower movements allow the camera more time to focus on the environment, thereby improving the quality of the 3D reconstruction. Another factor is that slower movements result in greater frame overlap, which increases the number of features that can be matched between frames. All this is visible in Figure 10.

In contrast, faster walking speeds, such as 0.164 m/s or above, significantly reduce the recording time and data size, making the process more efficient in terms of storage and computational load. However, this comes at the cost of increased motion blur and reduced frame overlap, which can compromise the accuracy of feature matching. For scenarios where reconstruction speed is prioritized over precision, faster speeds may still be acceptable, but they are not ideal for environments requiring high-detail reconstructions.

In summary, a slower filming speed increases the amount of data, enhances the quality of the data, and facilitates the matching of more features between frames, all of which contribute to higher-quality 3D reconstructions. It is recommended to use a walking speed of approximately 0.09 m/s to 0.12 m/s to balance clarity and efficiency.

### 3.3. Layering Technique for Enhanced 3D Reconstruction

The choice of using 1, 3, and 5 layers for the layering technique was guided by preliminary testing and practical constraints. A single-layer scan provides a baseline for comparison, representing the simplest and fastest capturing method. The three-layer configuration was chosen based on prior studies suggesting that capturing data at multiple heights enhances reconstruction fidelity without significantly increasing processing time. The five-layer configuration was included to test whether additional layers provide further improvements. Other configurations (e.g., 2, 4, or 6 layers) were initially considered, but early experiments indicated diminishing returns beyond three layers, as additional passes introduced excessive data without significantly enhancing reconstruction quality. Moreover, higher layer counts increased processing time and data complexity, making them less practical for real-world forensic applications.

Preliminary research underscores the significance of capturing an environment at varying heights to maximize data acquisition. This test aims to analyze the impact of using 1, 3, or 5 layers on the quality of 3D reconstruction. To evaluate the quality of each reconstruction, three reference images are utilized: the top of the table, the bottom of the table, and the ceiling. For an optimal 3D reconstruction, all these components should be clearly visible and of high quality.

Top of the Table:

- All three reconstructions (1, 3, and 5 layers) provided clear visibility of the tabletop.
- The scan with a single layer showed the sharpest 3D reconstruction with minimal noise.
- The scans with 3 and 5 layers, while acceptable, were slightly less sharp compared to the single-layer scan.

Bottom of the Table:

- The single-layer scan failed to capture the bottom of the table and the sides, including the legs of the chairs.
- Both the 3-layers and 5-layers scans successfully captured the bottom of the table and the chair legs.
- The 3-layers scan performed better in visualizing the sides and bottom of the table compared to the 5-layers scan.

Ceiling:

- The ceiling in the single-layer scan appeared pitch black due to the absence of data, which the AI algorithm filled with black.
- The difference between the 3-layers and 5-layers scans was minimal, with the 3-layers scan being slightly sharper.

**Figure 11:**
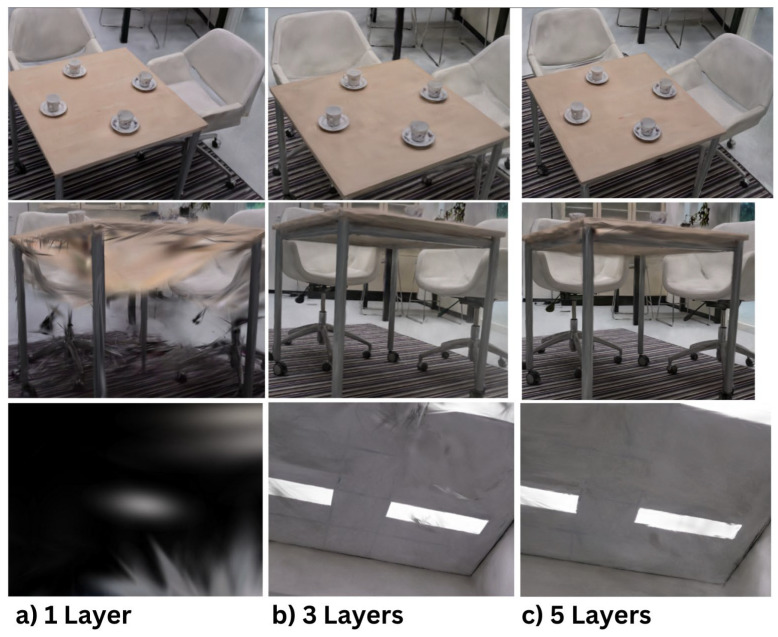
Layering Technique for Enhanced 3D Reconstruction (**a**) 1 Layer, (**b**) 3 Layers, (**c**) 5 Layers.

A single-layer scan is effective for capturing specific details, such as the surface of a table, but falls short when it comes to achieving a complete 3D reconstruction of an entire environment. The difference between the 3-layers and 5-layers scans was minimal, with the 3-layer scan often outperforming the 5-layer scan. This may be attributed to the 5-layers scan providing excessive data, complicating the algorithm’s ability to accurately align the frames. For this case, a 3-layers scan is sufficient. All of the above is visible in Figure 11.

### 3.4. Most Optimal Scan Method

#### 3.4.1. Summary of Methods

All the methods mentioned above have been tested. The chosen method within an environment significantly impacts the quality of the 3D reconstruction. Each scan was evaluated based on the amount of noise present, the level of detail achieved, and the number of splats (data density). The results varied across the different scans. The poorest quality was observed in Method 3, where the 3D reconstructed environment was barely recognizable, with a splats count of only 250,265 (rating 2). In contrast, Methods 5, 8, and 10 yielded excellent results, characterized by high detail, minimal noise, and high splats counts of 432,640, 489,000, and 474,357 (ratings 4 and 5), respectively. These three reconstructions will be discussed in detail in the next section.

The analysis primarily concludes that capturing the hallway, living room, and kitchen in a single video is ineffective. The algorithm receives an excessive amount of data, resulting in a poorly reconstructed model. Dividing the environment into multiple scans enhances the reconstruction quality, making it more detailed. The splats metric further supports this conclusion, as it quantifies the density of data points (splats) in the reconstruction. A low splats count, such as in Method 9 (212,211, rating 1), often indicates insufficient data density, leading to sparse or incomplete reconstructions. These models may exhibit noticeable gaps, lower texture quality, and reduced accuracy, making them unsuitable for applications requiring high fidelity, such as forensic investigations.

Conversely, a high splats count, as observed in Methods 5, 8, and 10 (432,640, 489,000, and 474,357 splats, ratings 4 and 5), reflects a dense and uniform distribution of data points. This results in smoother surfaces, reduced noise, and improved texture mapping quality, all of which contribute to more realistic and detailed 3D reconstructions. However, an excessively high splats count can also increase computational requirements, making the processing more resource intensive. This trade-off highlights the need to balance splats density with practical workflow considerations.

For the room used in these tests, segmenting it into a maximum of three segments ensures an optimal balance. This approach achieves sufficiently high splats counts for accurate reconstructions without overwhelming the reconstruction algorithm or requiring excessive computational resources. By leveraging the splats metric as an additional evaluation criterion, it becomes evident that methods focusing on segmentation and optimized data density produce the best results for detailed and reliable 3D reconstructions.

Reflecting on Method 5, scanning around a specific object (loop closures) proved beneficial. For instance, the scan around the coffee table produced a high-quality reconstruction. For scans 8 and 10, the environment was divided into three paths, which resulted in the best reconstructions. However, reconstructing the hallway remains difficult due to its height, narrowness, and large monotone surfaces, which complicate the reconstruction process. The analysis of all scanning methods is visible in Table 2.

#### 3.4.2. Method 5

**Figure 12:**
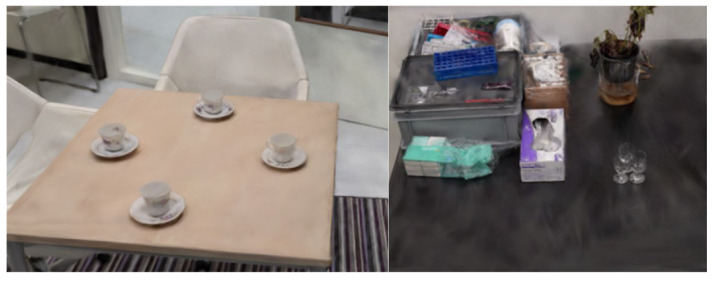
Method 5—Scanning kitchen and tabletop.

**Figure 13:**
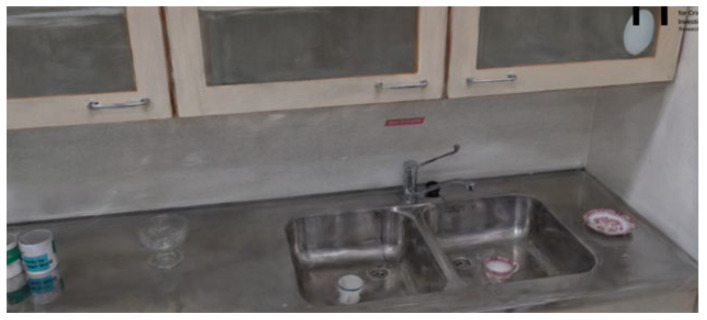
Method 5—Kitchen counter.

Method 5 yielded promising results. In this scan, each object or area was individually scanned, resulting in nine separate 3D reconstructions. Most of these individual reconstructions were high quality. Among the nine reconstructions, images were captured of the kitchen, the kitchen table, and the coffee table, as illustrated below. Figure 12 showcases the reconstruction of the kitchen table, where the table, coffee cups, and chairs are clearly visible with minimal noise in the 3D data. The objects on both the kitchen table and counter are well-defined and easily identifiable as shown in Figure 13.

Overall, scanning specific objects or zones led to more detailed and accurate 3D reconstructions, confirming that isolating individual areas improves reconstruction quality. However, one limitation is that current 3D reconstruction technology still requires manual work to merge separate scans. This means that each scan must be cropped, resized, and aligned to assemble a complete 3D reconstruction of an entire room. While larger zones can be connected, merging nine separate reconstructions would be time-consuming and challenging. Given the current limitations in splat file editing applications, this process is cumbersome and impractical for now. In summary, while focusing on individual zones enhances the quality of 3D reconstructions, this method is not yet feasible for whole-room reconstructions until more advanced algorithms or tools are developed to automate merging and clean up multiple 3D scans.

#### 3.4.3. Methods 8 and 10

**Figure 14:**
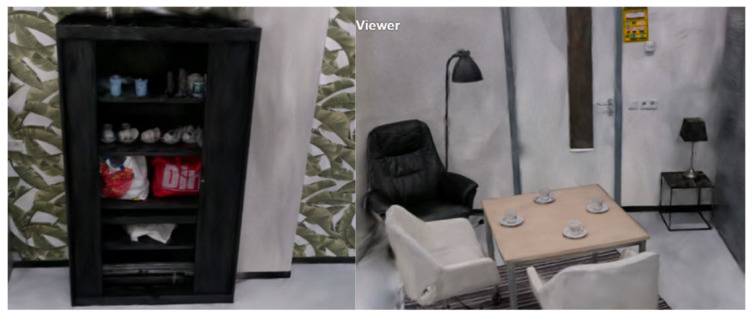
Results from Method 8 and Method 10.

**Figure 15:**
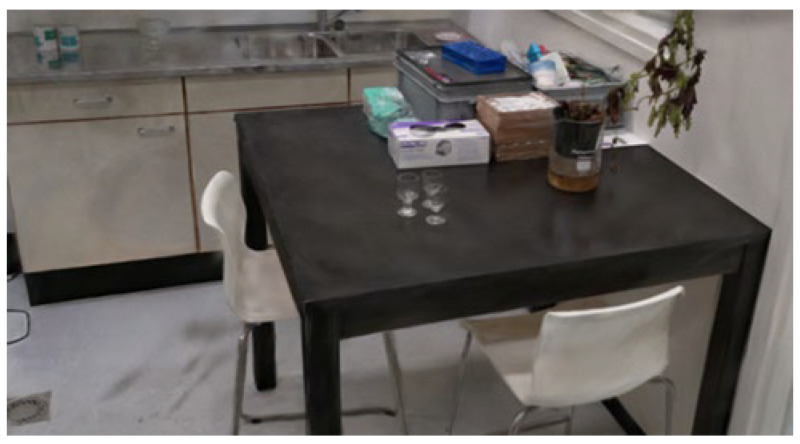
Tabletop results from Method 8 and Method 10.

Methods 8 and 10 are identical, with the exception that in Method 10, the area around the coffee table is extended to include the side of the black cabinet, resulting in a complete reconstruction of the cabinet. Figure 14 and Figure 15 show screenshots of the cabinet, kitchen, and coffee table, all of which are captured detailed. Some items even have readable text. However, there is still some noise in the reconstructions, although this can be removed with further processing.

In summary, Method 5 produced very detailed 3D reconstructions of individual objects and areas. However, due to the lack of effective techniques for stitching 3D reconstructions, this method is not suitable. It takes too much time to attach nine smaller 3D reconstructions to each other. Method 10 emerged as the best scan path. The general living/kitchen area was reconstructed well while maintaining an efficient workflow. However, despite its effectiveness in more complex areas, limitations persist, particularly in narrow or featureless spaces like hallways. The inability to effectively stitch reconstructions of individual objects into a cohesive whole reflects current technological constraints in handling multiple separate scans.

## 4. Discussion

This section evaluates the implications of the findings on capturing techniques for 3D reconstruction, emphasizing their practical applications, limitations, and alignment with the existing literature. By comparing the observed results with previous studies, this section identifies how specific capturing methods influence reconstruction quality, noise reduction, and detail accuracy. Additionally, it highlights the potential for refining current workflows, addresses the limitations imposed by technological constraints, and proposes avenues for future research to enhance the efficiency and adaptability of 3D reconstruction processes.

### 4.1. Comparison with the Literature

The importance of slow, deliberate camera movements in improving 3D reconstruction quality is well-documented. Zhang et al. [37] highlighted that optimized camera trajectories, such as those implemented in ROSEFusion, enhance feature matching and reduce noise artifacts even under dynamic conditions. Consistent with their findings, our study demonstrated that slower camera movements produced higher-quality reconstructions, with improved detail and reduced noise. This is attributed to increased frame overlap and better focus, aligning with the broader consensus in the literature.

Camera orientation also plays a pivotal role in the quality of 3D reconstructions. While portrait and landscape modes offer unique advantages, landscape orientation is generally preferred for its broader field of view and greater horizontal overlap. This observation is supported by principles in photogrammetry, which emphasize the importance of maximizing overlap for better feature recognition [38]. Our results reinforce this understanding, with landscape mode consistently yielding superior reconstructions compared to portrait mode, especially for horizontally expansive environments.

Reconstructing narrow or featureless spaces remains a persistent challenge. Lu et al. [39] identified that such environments hinder feature detection, impacting reconstruction accuracy. This was evident in our study, where hallways posed significant difficulties for reconstruction algorithms due to their monotone surfaces and lack of distinctive features. However, our segmentation-based methods, particularly Method 10, showed promise in mitigating these issues by dividing environments into manageable zones. This approach echoes similar strategies proposed in prior research but extends them by incorporating Gaussian Splatting techniques to further enhance reconstruction fidelity.

### 4.2. Potential Applications

The findings of this research have significant implications across multiple fields requiring high-quality 3D reconstructions. In crime scene investigations, accurate 3D models enhance evidence analysis and presentation by capturing spatial relationships and event sequences. Beyond forensics, these optimized methods support architecture, archaeology, and cultural heritage preservation, enabling precise, non-invasive documentation and restoration efforts while enhancing public engagement through virtual experiences.

In VR and AR, high-quality 3D models improve realism and immersion, benefiting training simulations, gaming, and education. By applying the recommended capturing techniques, developers can achieve clearer, more detailed models, broadening the applicability of 3D reconstruction technology across diverse professional domain

### 4.3. Limitations and Future Research

#### 4.3.1. Limitations

This study had several limitations. The experimental setup was constrained by the available space and objects, which may not be fully representative of typical crime scenes. Additionally, the inability to fully automate the merging of individual object scans into a single coherent model limited this study’s application to real-world crime scene reconstruction. This study was also limited by the specific technology and software used for 3D Gaussian Splattering (3D-GS), which may not have been the most advanced available at the time. The manual intervention required in aligning multiple scans remains a challenge, emphasizing the need for more sophisticated algorithms and tools for future studies.

Another limitation to consider is domain shift, which occurs when the developed methods are applied to environments significantly different from the controlled indoor settings used in this study. For example, outdoor crime scenes or disaster sites introduce unique challenges, such as dynamic lighting, larger spatial dimensions, and less predictable textures. These shifts could affect the performance of Gaussian Splatting and NeRF algorithms, necessitating domain-specific adaptations or improvements to maintain reconstruction quality. This will be a topic for future research. Initial experiments have already started, and the domain shift is considered.

While this study identified optimal filming techniques for indoor crime scene reconstructions, their generalizability to other environments, such as outdoor scenes or highly reflective surfaces, remains an open question. Outdoor crime scenes introduce additional challenges, including variable lighting, weather conditions, and larger spatial areas, which may impact the effectiveness of the scanning paths and layering techniques used in this study. Similarly, scenes with highly complex or featureless surfaces, such as reflective floors or monochromatic walls, could require adjustments to filming strategies to ensure sufficient feature matching. Future work should investigate how these techniques perform under different environmental conditions and whether adaptive filming strategies, such as exposure compensation or alternative scanning paths, are necessary to maintain reconstruction quality across diverse crime scene scenarios.

#### 4.3.2. Future Research

Future work should focus on overcoming the limitations of merging multiple scans into a cohesive whole, perhaps by advancing the software algorithms used in 3D reconstruction. Further exploration into using more advanced stabilizers or integrating markers within the scene could enhance the quality of the reconstructed models. Additionally, investigating how different lighting conditions or environments affect the 3D Gaussian Splattering process could broaden the applicability of this method in real-world crime scene reconstructions. There is also room for improvement in the reconstruction of narrow, featureless spaces like hallways, where more specialized techniques may be necessary.

**Figure 16:**
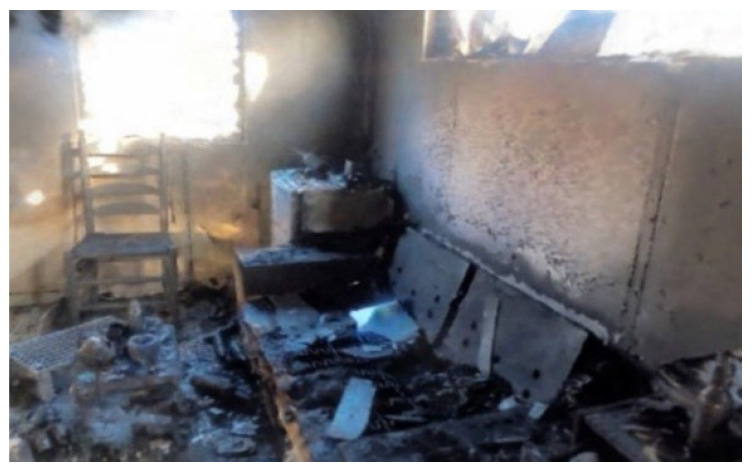
3D reconstruction of arson.

Expanding the scope of future work, we also plan to explore the application of these methods in diverse indoor and outdoor environments. While this study focused on simulated indoor scenarios for ease of replication, real-world environments such as abandoned buildings, train stations, or underground tunnels and outdoor areas present unique challenges, including variable lighting and complex spatial layouts [40]. For instance, outdoor environments may introduce domain shift due to differences in lighting conditions, object textures, and spatial dimensions, potentially affecting the performance of Gaussian Splatting and NeRF algorithms. Addressing these challenges through adaptive techniques, such as fine-tuning algorithms for specific domains or integrating more generalizable approaches, would significantly enhance the robustness of these methods. As an example, we are currently experimenting with 3D reconstructions of arson scenarios, which require capturing detailed and accurate representations of fire-damaged structures and debris. Initial work with cases of arson in both indoor and outdoor locations, as shown in Figure 16, demonstrates the need to test the adaptability and scalability of the proposed methods under diverse conditions. These efforts aim to ensure that the methods remain effective across varying scenarios, thereby expanding their utility in real-world forensic applications.

Future research could focus on integrating real-time feedback mechanisms during the filming process to enhance data acquisition. Such systems could identify areas with insufficient data coverage, where low frame density might lead to poor textures, artifacts, or cloud-like distortions in the final reconstruction. By providing immediate visual cues or alerts, operators could adapt their filming strategy on the spot, ensuring better coverage of critical areas before processing begins. This would help minimize gaps and inconsistencies, reducing the need for re-scanning and improving the overall quality of 3D reconstructions. Implementing this approach could lead to more efficient workflows, as adjustments could be made in real-time rather than relying solely on post-processing corrections

## 5. Conclusions

This study explored various parameters influencing the quality of 3D reconstructions in forensic contexts, focusing on capturing methods using handheld cameras in indoor environments. By systematically adjusting factors such as camera orientation, filming speed, layering techniques, and scanning paths, the research identified the most effective strategies for achieving detailed and accurate reconstructions of crime scenes.

The findings highlight the advantages of using a landscape orientation over a portrait mode, as it offers superior horizontal overlap, which is essential for aligning features effectively. This minimizes anomalies and ensures a more consistent reconstruction quality. Furthermore, the results demonstrated that slower camera movements significantly enhance the quality of reconstructions. The additional frames captured during slower movements allow for improved data quality and better feature matching, leading to reconstructions with higher detail and reduced noise.

In terms of layering, this study found that capturing the environment at three different heights provides an optimal balance between detail and processing efficiency. While additional layers occasionally introduced noise, the three-layer approach captured sufficient vertical data to reconstruct both the tops and bottoms of objects effectively. Scanning individual objects or areas, as seen in Method 5, produced high-quality reconstructions but was impractical due to the challenges of merging separate scans into a cohesive whole. Method 10, which employed segmented scanning with an additional loop for specific areas, emerged as the most practical approach, balancing detail and workflow efficiency. However, difficulties remained in reconstructing narrow, featureless spaces like hallways, underscoring limitations in current algorithms.

These findings provide valuable insights for forensic investigations, offering guidance on optimizing 3D reconstruction techniques with current technologies. While promising, this study also highlights the need for advancements in software to automate the merging of segmented scans into unified models. Future research could explore marker-based scanning techniques and more sophisticated reconstruction algorithms to address these challenges, enhancing the practical applicability of the proposed methods in real-world forensic scenarios.

By improving the quality and efficiency of 3D reconstructions, this study contributes to forensic science, architecture, and other fields requiring accurate spatial modeling, paving the way for more reliable and immersive applications of 3D technology.

## Figures and Tables

**Table 1:** Criteria for comparative analysis.

Criteria	1	2	3	4	5
Noise	There is too much noise present, and nothing can be seen	There is too much noise present, but the room is visible	There is some noise present; however, the outline of the room is still visible	Almost no noise is present, and the room is quite clear in visibility	There is no noise
Details (focus on blue bottle, walls, whiteboard, TV, and TCI coffee mug)	The reconstruction appears pixelated, yet it is discernible that an object should be present in that location	The reconstruction is pixelated, but it is still possible to discern the object type (e.g., table, chair, paper)	Identification of the object types is easily achievable	Capable of accurately identifying the object and providing brand information	Extremely detailed; there is no discernible difference between the model and the video
Splats (count)	Below 250,000 splats, very sparse data leading to low reconstruction fidelity	Between 250,000 and 300,000 splats, sparse data with visible gaps	Between 300,000 and 400,000 splats, moderate data density but some inconsistencies in texture mapping	Between 400,000 and 450,000 splats, high data density with uniform coverage and minimal artifacts	Above 450,000 splats, exceptionally dense data providing superior reconstruction and texture quality

**Table 2:** Comparison of Scan Methods.

Scan Methods	Noise	Details	Splats
1	3	4	4
2	2	3	3
3	2	2	2
4	4	3	5
5	4	4	4
6	3	3	3
7	2	4	2
8	4	4	5
9	2	3	1
10	4	5	5

## Data Availability

Data are contained within this article.

## References

[1] Mostafa Y.M., Al-Berry M.N., Shedeed H.A., Tolba M.F. (2023). Data Driven 3D Reconstruction from 2D Images: A Review. Lecture Notes on Data Engineering and Communications Technologies, Proceedings of the 8th International Conference on Advanced Intelligent Systems and Informatics 2022, Cairo, Egypt, 20–22 November 2022.

[2] Moons T., Van Gool L., Vergauwen M. (2008). 3D Reconstruction from Multiple Images Part 1: Principles. Found. Trends® Comput. Graph. Vis..

[3] Kang Z., Yang J., Yang Z., Cheng S. (2020). A Review of Techniques for 3D Reconstruction of Indoor Environments. ISPRS Int. J. Geo-Inf..

[4] Sharma O., Arora N., Sagar H. Image Acquisition for High Quality Architectural Reconstruction. Proceedings of the 45th Graphics Interface Conference.

[5] Apollonio F.I., Fantini F., Garagnani S., Gaiani M. (2021). A Photogrammetry-Based Workflow for the Accurate 3D Construction and Visualization of Museums Assets. Remote. Sens..

[6] Zhang C., Maga A.M. (2023). An Open-Source Photogrammetry Workflow for Reconstructing 3D Models. Integr. Org. Biol..

[7] Feng Y., Wu R., Liu X., Chen L. (2023). Three-Dimensional Reconstruction Based on Multiple Views of Structured Light Projectors and Point Cloud Registration Noise Removal for Fusion. Sensors.

[8] Fan J., Wang X., Zhou C., Zhang P., Jing F., Hou Z. (2023). Structured Light Vision 3-D Reconstruction System for Different Media Considering Refraction: Design, Modeling, and Calibration. IEEE/ASME Trans. Mechatron..

[9] Eltner A., Sofia G. (2020). Structure from motion photogrammetric technique. Dev. Earth Surf. Process..

[10] Wang X., Wang C., Liu B., Zhou X., Zhang L., Zheng J., Bai X. (2021). Multi-view stereo in the Deep Learning Era: A comprehensive review. Displays.

[11] Mildenhall B., Srinivasan P.P., Tancik M., Barron J.T., Ramamoorthi R., Ng R. (2020). NeRF: Representing Scenes as Neural Radiance Fields for View Synthesis. Lecture Notes in Computer Science (Including Subseries Lecture Notes in Artificial Intelligence and Lecture Notes in Bioinformatics), Proceedings of the 16th European Conference, Glasgow, UK, 23–28 August 2020.

[12] Kerbl B., Kopanas G., Leimkuehler T., Drettakis G. (2023). 3D Gaussian Splatting for Real-Time Radiance Field Rendering. ACM Trans. Graph..

[13] David A., Joy E., Kumar S., Bezaleel S.J. (2022). Integrating Virtual Reality with 3D Modeling for Interactive Architectural Visualization and Photorealistic Simulation: A Direction for Future Smart Construction Design Using a Game Engine. Lecture Notes in Networks and Systems, Proceedings of the Second International Conference on Image Processing and Capsule Networks: ICIPCN 2021, Bangkok, Thailand, 27–28 May 2021.

[14] Remondino F., El-Hakim S., Girardi S., Rizzi A., Benedetti S., Gonzo L. 3D Virtual Reconstruction and Visualization of Complex Architectures—The ‘3D-ARCH’ Project. www.stefanobenedetti.com.

[15] Bevilacqua M.G., Russo M., Giordano A., Spallone R. 3D Reconstruction, Digital Twinning, and Virtual Reality: Architectural Heritage Applications. Proceedings of the 2022 IEEE Conference on Virtual Reality and 3D User Interfaces Abstracts and Workshops (VRW).

[16] Gomes L., Bellon O.R.P., Silva L. (2014). 3D reconstruction methods for digital preservation of cultural heritage: A survey. Pattern Recognit. Lett..

[17] Cefalu A., Abdel-Wahab M., Peter M., Wenzel K., Fritsch D. Image based 3D Reconstruction in Cultural Heritage Preservation. Proceedings of the 10th International Conference on Informatics in Control, Automation and Robotics.

[18] Rodriguez-Garcia B., Guillen-Sanz H., Checa D., Bustillo A. (2024). A systematic review of virtual 3D reconstructions of Cultural Heritage in immersive Virtual Reality. Multimed. Tools Appl..

[19] Zhong C., Cheng S., Kasoar M., Arcucci R. (2023). Reduced-order digital twin and latent data assimilation for global wildfire prediction. Nat. Hazards Earth Syst. Sci..

[20] Cheng S., Liu C., Guo Y., Arcucci R. (2023). Efficient deep data assimilation with sparse observations and time-varying sensors. J. Comput. Phys..

[21] Nguyen-Phuoc T., Liu F., Xiao L. (2022). SneRF: Stylized Neural Implicit Representations for 3D Scenes. ACM Trans. Graph..

[22] Kulhanek J., Sattler T. (2023). Tetra-NeRF: Representing Neural Radiance Fields Using Tetrahedra. https://github.com/jkulhanek/tetra-nerf.

[23] Tancik M., Weber E., Ng E., Li R., Yi B., Wang T., Kristoffersen A., Austin J., Salahi K., Ahuja A. Nerfstudio: A Modular Framework for Neural Radiance Field Development. Proceedings of the SIGGRAPH 2023.

[24] Müller T., Evans A., Schied C., Keller A. (2022). Instant Neural Graphics Primitives with a Multiresolution Hash Encoding. ACM Trans. Graph..

[25] Liang R., Zhang J., Li H., Yang C., Guan Y., Vijaykumar N. (2022). SPIDR: SDF-Based Neural Point Fields for Illumination and Deformation. arXiv.

[26] Reiser C., Szeliski R., Verbin D., Srinivasan P., Mildenhall B., Geiger A., Barron J., Hedman P. (2023). MERF: Memory-Efficient Radiance Fields for Real-time View Synthesis in Unbounded Scenes. ACM Trans. Graph..

[27] Rangelov D., Knotter J., Miltchev R. (2024). 3D Reconstruction in Crime Scenes Investigation: Impacts, Benefits, and Limitations. Lecture Notes in Networks and Systems, Intelligent Systems and Applications, IntelliSys 2024, Amsterdam, The Netherlands, 5–6 September 2024.

[28] Rangelov D., Waanders S., Waanders K., van Keulen M., Miltchev R. (2024). Impact of Camera Settings on 3D Reconstruction Quality: Insights from NeRF and Gaussian Splatting. Sensors.

[29] Sony Sony Alpha 7C Full-Frame Mirrorless Camera—Black|ILCE7C. https://electronics.sony.com/imaging/interchangeable-lens-cameras/all-interchangeable-lens-cameras/p/ilce7c-b?srsltid=AfmBOoo5N6vG9O3tR3d9p7ZKy9YqWMPZSzdnQnfZjfl4XP9WE2vRx1bz.

[30] Sigma 14mm F1.4 DG DN|Art|Lenses|SIGMA Corporation. https://www.sigma-global.com/en/lenses/a023_14_14/.

[31] DJI DJI RS 4—Gripping Storytelling—DJI. https://www.dji.com/bg/rs-4.

[32] Jawset Jawset Postshot. https://www.jawset.com/.

[33] Tosi F., Zhang Y., Gong Z., Sandström E., Mattoccia S., Oswald M.R., Poggi M. (2024). How NeRFs and 3D Gaussian Splatting are Reshaping SLAM: A Survey. arXiv.

[34] Yurkova K. A Comprehensive Overview of Gaussian Splatting|Towards Data Science. https://medium.com/towards-data-science/a-comprehensive-overview-of-gaussian-splatting-e7d570081362.

[35] Canon What Is Aperture Photography?|Canon U.S.A., Inc. https://www.usa.canon.com/learning/training-articles/training-articles-list/what-is-aperture.

[36] Garmin fēnix® 6 Pro Solar|Multisport Solar Watch. https://www.garmin.com/en-US/p/702902.

[37] Zhang J., Zhu C., Zheng L., Xu K. (2021). ROSEFusion: Random Optimization for Online Dense Reconstruction under Fast Camera Motion. ACM Trans. Graph..

[38] Lefcourt D. Portrait vs Landscape Orientation in Photography (Which Is Better?)—Lefcourt Photography. https://www.lefcourtphotography.com/portrait-vs-landscape-orientation-in-photography-which-is-better.

[39] Lu F., Zhou B., Zhang Y., Zhao Q. (2018). Real-time 3D scene reconstruction with dynamically moving object using a single depth camera. Vis. Comput..

[40] Fang X., Easwaran A., Genest B., Suganthan P.N. (2024). Your Data Is Not Perfect: Towards Cross-Domain Out-of-Distribution Detection in Class-Imbalanced Data. arXiv.

